# Experience of chronic noncommunicable disease in people living with HIV: a systematic review and meta-aggregation of qualitative studies

**DOI:** 10.1186/s12889-021-11698-5

**Published:** 2021-09-10

**Authors:** Zhongfang Yang, Zheng Zhu, Lucylynn Lizarondo, Weijie Xing, Shuyu Han, Hui Hu, Yan Hu, Bei Wu

**Affiliations:** 1 Fudan University School of Nursing, Shanghai, China; 2 Fudan University Centre for Evidence-based Nursing: A Joanna Briggs Institute Centre of Excellence, Shanghai, China; 3 Joanna Briggs Institute, University of Adelaide, Adelaide, South Australia Australia; 4 Department of Epidemiology, College of Public Health and Health Professions & College of Medicine, University of Florida, Gainesville, FL USA; 5 NYU Rory Meyers College of Nursing, New York City, New York USA

**Keywords:** HIV/AIDS, Chronic noncommunicable disease, Comorbidity, Systematic review

## Abstract

**Background:**

An increasing number of people living with HIV (PLWH) have had chronic noncommunicable diseases (NCDs) over the last 5 years. However, robust evidence regarding the perception and challenges of having NCDs among PLWH is limited. Therefore, this study aimed to synthesize qualitative evidence regarding the experiences of PLWH with NCDs.

**Methods:**

We used a meta-aggregation approach to synthesize qualitative studies. Peer-reviewed and gray literature published in English and Chinese from 1996 to November 2020 was searched using electronic databases. Two reviewers independently appraised the methodological quality and extracted data from the included studies. The Joanna Briggs Institute (JBI) meta-aggregation approach was used to synthesize the findings.

**Results:**

In total, 10,594 studies were identified in the initial database search. Fourteen eligible studies were included in the meta-synthesis. Among these studies, nine synthesized findings regarding the following topics were identified: fragmented healthcare systems, care continuity, manifestations of multiple conditions, financial hardship, stigma and discrimination, polypharmacy burden and adherence, reciprocal relationships between HIV and NCDs, and coping strategies.

**Conclusions:**

In recent years, attempts have been made to institutionalize NCD preventive and control services in HIV long-term care. However, considering the growing problem of HIV and NCD comorbidity globally, integrated primary health care systems are needed to address the problems of PLWH with NCDs. Healthcare professionals should help PLWH develop strategies to better monitor their polypharmacy burden and adherence, stigma and discrimination, financial hardship, and manifestations of multiple conditions to achieve high levels of care continuity.

**Supplementary Information:**

The online version contains supplementary material available at 10.1186/s12889-021-11698-5.

## Introduction

The life expectancy of people living with HIV (PLWH) has been steadily increasing due to the introduction of antiretroviral therapy (ART) in 1996. As reported by the Joint United Nations Programme on HIV/AIDS (UNAIDS) Coordinating Board, the proportion of PLWH aged 50 years and above will increase from 47% in 2020 to 95% in 2030 [1]. Studies concerning HIV and aging suggest that a growing number of PLWH face aging and age-related issues, including chronic noncommunicable diseases (NCDs) [2, 3]. NCDs are defined by the World Health Organization as noninfectious health conditions such as cardiovascular disease, cancer, chronic respiratory disease, and diabetes [4].

Although PLWH may suffer from the same NCDs as older individuals in the general population, the prevalence of NCDs is greater among PLWH [5, 6]. Research interest in chronic conditions among PLWH has increased because of the growing number of PLWH with NCDs over the last 5 years [7]. Xu and colleagues conducted a systematic review of 49 published studies and found that the prevalence of hypertension among PLWH was 25.2%, which is consistent with a WHO report [8]. Daultrey and colleagues’ systematic review revealed that among the 45 included studies, the prevalence of diabetes among PLWH ranged from 1.3 to 26% based on different diagnostic criteria [9]. The prevalence of chronic obstructive pulmonary disease, chronic kidney disease, and cardiovascular disease was 10.5, 4.8–6.4%, and 61.8%, respectively [10–12]. Woldesemayat’s study surveying 380 PLWH in Ethiopia showed that 51.6% of the participants had at least one type of NCD and that 8.9% of the participants had more than two types of NCDs [13]. Zhu’s studies also reported that PLWH in China suffered a mean of 7 symptoms from both HIV and NCDs [14, 15].

An in-depth understanding of how PLWH with NCDs cope with challenges in daily life has been highlighted in the recent literature as a knowledge foundation for developing effective professional care services and self-management strategies for PLWH [16]. The experiences of meeting these challenges among PLWH have been qualitatively reported in the recent literature. Abele conducted in-depth interviews with PLWH aged 50 years and older with comorbidities to describe the phenomenon of living with HIV and comorbidities in an aging PLWH population in the US [17]. Bosire and colleagues conducted a case study of three women with PLWH with breast cancer and hypertension to explore how women in South Africa coped with multiple chronic diseases and HIV [18].

Although qualitative studies have gathered information describing the experience of NCDs among PLWH, more research is needed to generate more robust evidence regarding the perception and challenges of having NCDs among PLWH by integrating information regarding various experiences of PLWH from various qualitative studies. To the best of our knowledge, no systematic review specific to the experiences of PLWH with NCDs has been published. The aim of this review is to synthesize qualitative evidence of the experiences of PLWH with NCDs. The Joanna Briggs Institute (JBI) Reviewer’s Manual and Preferred Reporting Items for Systematic Reviews and Meta-Analyses (PRISMA) statement were employed to guide the methodology and reporting of this systematic review [19, 20].

## Methods

### Search strategy

A three-step comprehensive search strategy was adopted to find both peer-reviewed and gray literature in English and Chinese. An initial search was conducted in PubMed to locate a small number of relevant studies to construct a more comprehensive search strategy. In the comprehensive search step, PubMed, MEDLINE (Ovid), Embase (Ovid), CINHAL (EBSCO), ProQuest Dissertations and Theses, Web of Science, Wangfang (Chinese), CNKI (Chinese), Google Scholar, and Baidu Scholar (Chinese) were searched. The search time frame was set from January 1996 to November 2020. The starting point was set in 1996 because antiretroviral therapy started to be applied among PLWH in 1996. In PubMed, we combined the following free words: ([hiv OR hiv infect* OR AIDS OR “human immunodeficiency virus”] AND [comorbid* OR comorbid* OR multimorbid* OR multi-morbid* OR multidisease? OR multi-disease?]) and MeSH terms ([“HIV” OR “Acquired Immunodeficiency Syndrome”] OR [“Comorbidity” OR “Multiple Chronic Conditions” OR “NCDs (see Additional file 1)”]). The search strategies used in all databases are available in Additional file 1. Finally, an additional search was conducted by hand-searching the reference lists of all included papers eligible for inclusion.

### Inclusion and exclusion criteria

Studies were included if they 1) aimed to explore the experience of having any types of chronic NCDs among PLWH with chronic NCDs, including diabetes, hypertension, heart diseases, chronic respiratory conditions, arthritis, etc.; 2) included PLWH aged 18 and over; 3) used qualitative methodologies, including phenomenology, ground theory, case study, ethnography, narrative study, etc. (mixed-method studies were also considered if they provided the participants’ quotations); and 4) were published in English or Chinese. Studies conducted in any settings or contexts were considered.

### Study screening and selection

All identified records retrieved from the databases were imported into EndNote (Clarivate Analytics, PA, USA). After the removal of duplicates, two reviewers (ZZ & ZY) independently reviewed the titles, abstracts, and full texts and determined whether the articles could be included according to the inclusion and exclusion criteria. Any disagreements were resolved by a third reviewer (YH). The reasons for exclusion after the full-text screening were documented.

### Quality appraisal

We used the JBI Critical Appraisal Checklist for Qualitative Research to assess the methodological quality of the included studies [20]. Two reviewers (ZY & ZZ) independently appraised the studies. Any disagreements between the two reviewers were resolved by a third reviewer (YH) to obtain consensus.

### Data extraction

Data regarding the authors, year of publication, country, design, methods of data collection, phenomenon of interest, characteristics of the participants, and main findings were extracted independently by two reviewers (ZZ & ZY) using an extraction template. Any discrepancies were resolved by a third reviewer (SH). The results are presented in narrative and tabular formats.

### Data synthesis

We conducted a meta-aggregation using the JBI approach to synthesize the findings reported in the included studies. The data synthesis process involved four steps. 1) The participants’ quotations and authors’ themes and subthemes relevant to our aim were extracted by one reviewer (ZZ) and double-checked by another reviewer (ZY). 2) Two reviewers (ZZ & ZY) independently rated the credibility of each finding to represent congruity between the findings and quotations. There were three levels of credibility as follows: unequivocal, credible, and unsupported. If more than one quotation described the same findings, we chose the quotation with the highest level of credibility (unequivocal > credible > unsupported). 3) Only findings rated as unequivocal and credible were grouped into categories. Any findings rated as unsupported were not included in further analysis. 4) The findings were grouped into categories based on similarities in meanings, and the categories were further integrated into the nine synthesized findings. All authors agreed upon the presented categories and synthesized findings and reached consensus after discussing the discrepancies during the review.

### Confidence in the findings

We used the Confidence in the Output of Qualitative Research Synthesis (ConQual) approach to guide us in assessing the level of confidence in the synthesized findings. The confidence levels included high, moderate, low, and very low and were determined based on the dependability and credibility of each included study. The level of dependability of each study was established based on five questions from the JBI Critical Appraisal Checklist for Qualitative Research (C2, C3, C4, C6, and C7). If four or five of the responses to these questions were yes, the dependability level of these synthesized findings remained at the currently appraised level. If two or three of the responses were yes, the dependability level was downgraded by one level. Otherwise, the dependability level was downgraded by two levels. The credibility level of the synthesized findings was determined by the credibility levels of the findings. For mixed unequivocal/credible (U/C) findings, the credibility of the synthesized findings was downgraded by one level. Otherwise, the credibility remained at the currently appraised level.

## Results

### Literature search

**Fig. 1:**
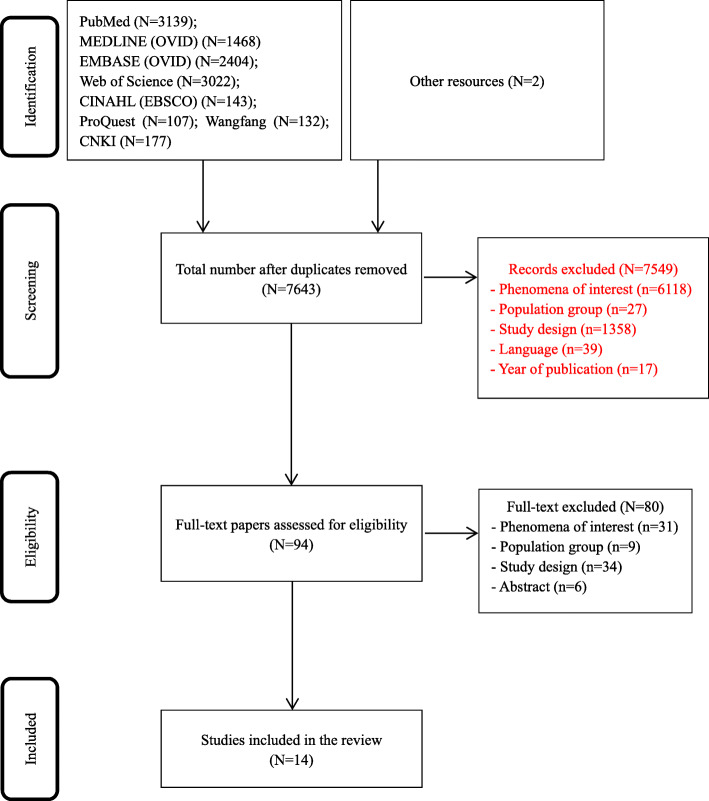
Flowchart of the identification and selection of studies

As shown in Fig. 1, in total, 10,594 studies were identified in the database search. After the removal of duplicates and the screening of the titles and abstracts, 94 studies were selected for full-text screening. In total, 14 eligible studies were included in the meta-synthesis [17, 18, 21–32].

### Quality appraisal

The results of the quality appraisal of the included studies are presented in Table 1. Only two papers addressed question C6 by using a cultural lens to describe the research findings and interpreted the findings within a social context [18, 25]. Four studies described the influence of the researchers on the research [17, 18, 25, 27]. Three studies did not provide sufficient details regarding ethical approval [26, 31, 32].

**Table 1:** Results of the quality appraisal^a^

	C1	C2	C3	C4	C5	C6	C7	C8	C9	C10
Abele [17] (2018)	U	Y	Y	Y	Y	U	Y	Y	Y	Y
Bosire et al. [18] (2020)	U	Y	Y	Y	Y	Y	U	Y	Y	Y
Bosire [21] (2020)	U	Y	Y	Y	Y	U	U	Y	Y	Y
Corrigan et al. [22] (2020)	U	Y	Y	Y	Y	U	U	Y	Y	Y
Gonah et al. [23] (2020)	U	Y	Y	Y	Y	U	U	Y	Y	Y
Hing et al. [24] (2020)	U	Y	Y	Y	Y	U	Y	Y	Y	Y
Mendenhall et al. [25] (2019)	U	Y	Y	Y	Y	Y	Y	Y	Y	Y
Monroe et al. [26] (2013)	U	Y	Y	Y	Y	U	U	Y	U	Y
Morgan et al. [27] (2018)	U	Y	Y	Y	Y	U	Y	Y	Y	Y
Muiruri et al. [28] (2020)	U	Y	Y	Y	Y	U	U	Y	Y	Y
Peer et al. [29] (2020)	U	Y	Y	Y	Y	U	U	Y	Y	Y
Simonik et al. [30] (2016)	U	Y	Y	Y	Y	U	U	Y	Y	Y
Slomka et al. [31] (2017)	U	Y	Y	Y	Y	U	U	Y	U	Y
Warren-Jeanpiere et al. [32] (2014)	U	Y	Y	Y	Y	U	U	Y	U	Y

^a^C1 = Congruity between the stated philosophical perspective and the research methodology

C2 = Congruity between the research methodology and the research question or objectives

C3 = Congruity between the research methodology and the methods used to collect the data

C4 = Congruity between the research methodology and the representation and analysis of the data

C5 = Congruity between the research methodology and the interpretation of the results

C6 = Statement locating the researcher culturally or theoretically

C7 = Statement of the influence of the researcher on the research

C8 = Representation of the participants and their voices

C9 = Ethical approval by an appropriate body

C10 = Relationship between the conclusions and analysis or interpretation of the data

*Y* Yes, *N* No, *U* Unclear, *NA* Not applicable

### Study description

Table 2 summarizes the characteristics of the included studies. All included studies were published after 2013. The studies were conducted in the following five different countries: the United States (*n* = 6) [14, 22, 26, 28, 31, 32], South Africa (*n* = 5) [18, 21, 23, 25, 29], Canada (*n* = 1) [30], Malawi (*n* = 1) [24], and Ghana (*n* = 1) [27]. Six studies used phenomenological designs [17, 24–27, 31, 32], and four used descriptive designs [22, 23, 28, 30]. The other designs included case studies [18], ethnographic studies [21], and mixed method studies [29]. Eleven studies used unstructured or semistructured interviews to collect data [17, 18, 21–25, 27–30], six studies used focus group interviews [23, 26, 28, 29, 31, 32], and three studies used both approaches [23, 28, 29]. The total number of PLWH included in this systematic review was 438, and the sample sizes of the individual studies ranged from 3 [18] to 80 participants [25]. Hypertension was the most reported NCD. Seven studies included PLWH with hypertension [17, 21, 22, 24, 26, 28, 29]. Seven studies included PLWH with various chronic conditions, including cancer, diabetes, arthritis, chronic pain, depression, and anxiety [18, 22, 25, 27, 30–32].

**Table 2:** Characteristics of the studies

Study (year), Country	Design (data collection method)	Phenomenon of interest	Recruitment and participants	Main findings
Abele [17] (2018), US	Phenomenological study (in-depth interviews)	To explore the experience of PLWH aged over 50 living with HIV and comorbidities.	Participants: 10 PLWH aged over 50; Mean age: 60.5 yrs.; Male: 80%; Comorbidities: at least 1 type of comorbidity; Race: not specific; Participants were recruited from an HIV agency in the US and an online HIV community.	Three themes were identified: 1) from fear of the unknown to ownership; 2) from ownership to survival; and 3) the constant struggle of surviving with HIV.
Bosire et al. [18] (2020), South Africa	Case study (narrative interviews)	To explore how women address multiple chronic and infectious diseases.	Participants: 3 women PLWH; Age: 30–45 yrs.; Male: 0%; Race: Black; Comorbidities: two participants living with breast cancer, hypertension, and HIV; Patients were recruited from a cancer study in South Africa.	One theme was identified in PLWH: social and economics of family.
Bosire [21] (2020), South Africa	Ethnographic study (narrative interviews)	To describe the experiences of PLWH seeking care for comorbid HIV and diabetes in a hospital in South Africa.	Participants: 15 PLWH; Age: 40–70 yrs.; Male: 46.6%; Race: Black; Comorbidity: Type 2 diabetes; Economic status: more than half of PLWH were unemployed with $60.72–$121.45 income per month; Participants were recruited from one diabetes clinic at a tertiary hospital in South Africa.	Two themes were identified: 1) patients’ experiences accessing chronic care and 2) patients’ experiences of self-management at home.
Corrigan et al. [22] (2020), US	Descriptive study (semistructured interviews)	To explore the perception of cancer care experience among PLWH.	Participants: 27 PLWH; Median age: 56 yrs.; Male: 81.5%; Race: 70.4% were African American, 25.9% were Caucasian, and 3.7% were Hispanic/Latino; Comorbidity: cancer; Economic status: median annual household income was $24,000; Participants at different stages in the cancer care process were recruited by using DEDUCE software and electronic medical records.	Eight types of barriers to care were identified: 1) side effects from treatment; 2) stigma; 3) accessibility issues; 4) financial burden of cancer treatment; 5) emotional and mental health difficulties; 6) family or personal issues; 7) mistrust in providers; and 8) fear of cancer diagnosis and treatments.
Gonah et al. [23] (2020), South Africa	Descriptive study (key informant interviews and focus group discussions)	To identify the challenges and ways of coping of PLWH with comorbid hypertension and diabetes mellitus accessing care.	Participants: 8 health managers and 72 PLWH; Age: 50% aged 30–49 yrs. and 50% aged > 50 yrs.; Male: 33.3%; Comorbidities: hypertension and/or diabetes mellitus); All participants were recruited from six ART sites in South Africa.	Three themes were identified: 1) management of hypertension and diabetes mellitus; 2) capacity to screen hypertension and diabetes mellitus; and 3) capacity to treat hypertension and diabetes mellitus.
Hing et al. [24] (2019), Malawi	Phenomenological study (semistructured interviews)	To explore the experience and perception of HIV and hypertension among PLWH.	Participants: 75 PLWH; Median age: 53 yrs.; Male: 36%; Comorbidity: hypertension; Participants were recruited from an HIV treatment site in Malawi.	Six themes were identified: 1) perceived susceptibility to comorbidities; 2) perceived severity of comorbidities; 3) perceived benefits of controlling comorbidities; 4) perceived barriers to controlling comorbidities; 5) perceived self-efficacy in controlling comorbidities; and 6) cues to action for controlling comorbidities.
Mendenhall et al. [25] (2019), South Africa	Phenomenological study (narrative interviews)	To explore how Black South Africans perceive and experience multiple concurrent medical conditions.	Participants: 80 participants with multimorbidities; Mean age: 56 yrs.; Male: 37.5%; Economic status: median monthly income was $196; Comorbidities: various types of comorbidities, including hypertension, HIV, obesity, diabetes, tuberculosis, arthritis, chronic pain, depression, and anxiety; Participants were recruited from two hospitals in South Africa.	Five themes regarding HIV and comorbidities were identified: 1) complexity of multimorbidities; 2) defining sickness and health; 3) understanding cancer; 4) differentiating between conditions; and 5) managing multiple conditions.
Monroe et al. [26] (2013), US	Phenomenological study (focus group)	To explore perspectives of living with HIV and diabetes mellitus or hypertension among PLWH and the factors affecting their medication adherence.	Participants: 35 PLWH; Mean age: 51 yrs.; Male: 54%; Race: Black 94%; Comorbidity: diabetes or hypertension; Participants were recruited through self-referrals from flyers and through referrals from medical providers in one HIV clinic in the US.	Four themes were identified: 1) comorbidities generate concern and frustration; 2) understanding health conditions and medications promotes adherence; 3) simpler regimens with fewer side effects promote adherence; and 4) untreated substance abuse and mental health issues hinder adherence.
Morgan et al. [27] (2018), Ghana	Phenomenological study (semistructured interviews)	To explore the perceptions and experiences of women living with multimorbidity in Ghana.	Participants: 20 women participants with multimorbidity Mean age: 56 yrs.; Male: 0%; Economic status: 60% were employed; Comorbidity: HIV was the most common condition among the participants; Participants were recruited from three polyclinics in Ghana.	Four themes emerged: 1) the influences on patients’ health experience; 2) seeking care and the responsiveness of the health care system; 3) how patients manage health care demands; and 4) outcomes due to health.
Muiruri et al. [28] (2020), US	Descriptive study (semistructured interviews and focus group discussion)	To identify the factors associated with adherence to cardiovascular disease (CVD) medications among PLWH with viral suppression.	Participants: 51 PLWH taking ART and CVD medications Mean age: 57 yrs.; Male: 66.7%; Race: Africa American 60.8%; Economic status: Monthly income $983; Participants were recruited from three hospitals in the US.	Three main themes were identified: 1) CVD prevention preferences and practices; 2) impact of long-term HIV care on adherence to CVD medication; and 3) factors motivating the adoption of and adherence to heart healthy behaviors
Peer et al. [29] (2020), South Africa	Mix-methods study (focus group and in-depth and semistructured interviews)	To explore the perceptions and experiences of PLWH with hypertension and identify their healthcare providers’ experiences interacting with patients with multiple comorbidities in South Africa.	Participants: 55 PLWH, 11 clinicians, 10 specialized nursing professionals, and 12 lay counselors; Comorbidity: hypertension; Participants were recruited from 17 public healthcare facilities in South Africa.	Four themes were revealed: 1) patient resources and priorities for HIV management; 2) clinical resources and priorities for HIV management; 3) patient resources and priorities for comorbid NCD management; and 4) clinical resources and priorities for comorbid NCD management.
Simonik et al. [30] (2016), Canada	Descriptive study (semistructured interviews)	To explore readiness to engage in exercise among PLWH with multimorbidity.	Participants: 14 PLWH (HIV and other morbidities); Median age: 50 yrs.; Male: 64.3%; Race: Caucasian 35.7%, Aboriginal/first nation 14.3%, other 21.4%, and not identified 28.6%; Comorbidities: asthma, cancer, eye disorder, hepatitis C, mental health conditions, muscle pain, joint pain, hypertension, peripheral neuropathy, arrhythmia, and neurocognitive decline; Participants were recruited from a specialty hospital in Canada.	Readiness was influenced by 1) physical impairments; 2) mental health challenges; and 3) uncertainty due to HIV and concurrent health conditions. Subfactors that influenced readiness to exercise included 1) social support; 2) perceptions and beliefs; 3) past experience with exercise; and 4) accessibility.
Slomka et al. [31] (2017), US	Phenomenological study (focus group interviews)	To explore the lived experiences of multimorbidity among PLWH.	Participants: 22 PLWH with at least one other chronic condition; Mean age: 51 yrs.; Male: 73%; Race: Africa American 81.8%; Comorbidities: hypertension, heart disease, heart failure, kidney disease, hepatitis C, liver disease, mental health conditions, diabetes; Participants were recruited from a large specialty HIV clinic in the US.	Four themes were identified: 1) HIV as a background for other chronic conditions; 2) managing medications and provider interactions; 3) coping with future health care needs; and 4) stigmatized social environment of multimorbidity with HIV.
Warren-Jeanpiere et al. [32] (2014), US	Phenomenological study (focus group interview)	To explore how the age, identity, comorbidities, social responsibilities, and relationship status of older HIV-positive African American women influence their HIV self-management.	Participants: 23 PLWH; Mean age: 57 yrs.; Male: 0%; Race: Africa American 100%; Comorbidities: arthritis, high blood pressure, hepatitis, cancer, depression, heart disease, diabetes; Participants were recruited from one hospital in the US.	Four themes were identified: 1) “Taking it one day at a time”; 2) “Age ain’t nothing but a number”; 3) “Forget the single life”; and 4) “Daily life struggles”.

### Main findings

Table 3 shows an overview of the synthesized findings. In these 14 articles, we identified 155 findings, 37 categories, and 9 synthesized findings. Overall, the level of credibility of the findings was not high. Among all findings, we rated 146 findings as unequivocal and 9 findings as credible.

**Table 3:** Synthesized findings

Findings	Categories	Synthesized Findings
Difficulty finding a parking place (U) [22]	Limited access to HIV-related healthcare services	1. Although the current healthcare systems have many barriers preventing PLWH with NCDs from accessing both HIV and NCD care, they can receive resources and knowledge through healthcare providers.
Challenges with transport costs (U) [21, 23, 30]		
Medication shortage (U) [23, 27]		
Long waiting times at healthcare centers (U) [29]		
Limited income (U) [23, 30]		
Mobility restrictions stemming from HIV (U) [30]		
Challenges with physical disability (U) [30]		
Ability of current healthcare system to meet PLWH needs (U) [31]		
Fear of being stigmatized for having HIV (U) [29]		
Unavailability of screening machines for hypertension and diabetes (U) [23]	Barriers to accessing healthcare for NCDs	
Mobility restrictions stemming from other chronic conditions (U) [30]		
Unavailability of medication for hypertension (U) [23]		
Lack of care continuity (U) [29]		
Insufficient care for chronic diseases (U) [29]		
Lack of familiarity with the situations of PLWH among healthcare providers from NCD departments (U) [31]		
Simultaneous acquisition of knowledge regarding the disease process and social support (U) [17]	Good aspects of the current healthcare systems	
Utilization of the available resources to avoid isolation and depression (U) [17]		
Satisfaction with providers (U) [17, 22]		
Likely elimination of the stigma attached to the ARV clinic through the provision of integrated care through general systems (U) [29]		
Understanding the consequence of nonadherence to treatment (U) [26]	Individual factors affecting care continuity and treatment adherence	2. The factors that can affect care continuity and treatment adherence for both HIV and NCDs among PLWH include the individual physical, mental, and financial statuses of PLWH; their relationships with clinicians; and fragmented healthcare systems.
Linking symptoms to not taking medication (U) [24, 26]		
Active engagement in treatment (U) [30]		
Substance abuse (U) [26]		
Mental issues (U) [26]		
Ease of obtaining prescriptions (U) [23, 26, 27]		
Health insurance scheme (C) [27]		
Financial issues (U) [27]		
Encouragement to continue treatment due to improved symptoms (U) [27]		
Fragmented and uncoordinated HIV care and chronic care (U) [21, 22, 29, 31]	Fragmented healthcare systems	
Distance between the tertiary hospital and HIV clinics (U) [21]		
Effect of mistrust of providers on treatment adherence (U) [22]	Patient-clinician partnership	
Good partnership with clinicians (U) [17, 22]		
HIV being beyond the scope of practice of generalists (U) [17, 22, 29]		
Lack of communication (U) [22]		
Effect of appearance on self-esteem (U) [17]	Appearance	3. PLWH with NCDs have long-term physical and psychological manifestations of disease. Psychological manifestations are severe and prevalent when PLWH are informed that they have NCDs. Some changes, including changes in appearance and sexual function, can affect individuals’ daily lives, especially among women living with HIV and NCDs.
Dental issues (C) [17]		
Importance of maintenance physical appearance for women (U) [32]		
Fear, anger, and rejection (U) [17]	Psychological manifestations	
Stress and vulnerability due to the management of multiple conditions (U) [21]		
Similarity between reactions to a cancer diagnosis and initial reactions to the HIV diagnosis (U) [22]		
Fear of cancer diagnosis and treatments (U) [22]		
Mental health treatment (U) [22]		
Initial difficulty in accepting the diagnosis of hypertension (U) [24]		
Negative emotions upon being informed of having multimorbidities (U) [29]		
Effect on women, dyspareunia (U) [17]	Sexual function	
Effect on one’s life (U) [17]		
Extreme fatigue, neuropathy, and memory loss (U) [17]	Physical manifestations	
Disability (U) [17]		
Symptoms disrupting everyday life (U) [27]		
Lack of an assumption that any new symptoms are related to HIV (U) [31]		
Effects on employment (U) [17, 18]	Social consequence of having NCDs	4. Having more chronic conditions implies more medical costs for PLWH. The more severe the financial hardship of PLWH, the more negatively it affects their employment and family relationships.
Effects on relations between family members (U) [18]		
Low confidence, even futility (U) [24]		
Numerous hospital appointments (U) [18]		
Struggles to obtain medication (U) [17, 23]	Financial and insurance issues	
Battles with their insurance (U) [17, 32]		
Reliance on a minimal state disability grant (U) [18]		
Financial burden of HIV and cancer treatment (U) [22, 23, 27, 32]		
Comparison of free ART with hypertension medication (U) [24]		
Lack of income (U) [32]		
Fear of rejection before acceptance (U) [17]	Double stigma	5. PLWH with NCDs experience double stigma toward their HIV and chronic conditions, which may exacerbate their perceived discrimination and lead to social and physical isolation.
Courtesy stigma (U) [18]		
Discrimination leading to social and physical isolation (U) [18]		
Personal insecurity within one’s home (U) [18]		
Disowning or violent behavior toward the sick person (U) [18]		
HIV stigmatization (U) [17, 18, 22, 27, 29]		
Fear of stigma or addition of stress to relationships (U) [17, 27]		
Stigma among support system and providers (U) [22]		
New form of stigma toward cancer (U) [25]		
Stigmatized social environment of multimorbidity with HIV (U) [31]		
Waiting for as long as possible before sharing one’s diagnosis with family members (U) [17]	Disclosure	
Openly sharing one’s diagnosis with friends but ultimately losing them (U) [17]		
Pill burden for managing both conditions (U) [24]	Pill burden	6. PLWH with NCDs have high levels of polypharmacy burden for both ART and other medications. This population has difficulty maintaining high medication adherence due to medication fatigue, side effects, and the large numbers of pills to be taken. PLWH with NCDs may spontaneously use some strategies individually or collectively to achieve high levels of medication adherence.
Taking pills daily as a form of survivorship (U) [17]	Polypharmacy adherence	
Taking multiple drugs as compensation for missed doses (U) [23]		
Side effects of hypertension medication as an impediment to adherence (C) [24]		
Simpler regimens with fewer side effects promoting adherence (U) [26]		
Medication fatigue (U) [32]		
Difficulty keeping up with the management regimens for their other comorbidities (U) [32]		
Side effects of ART (U) [17, 28, 31]	Side effects	
Side effects of hypertension medication as an impediment to adherence (C) [24, 28]		
Side effects of medication interaction (U) [31]		
Symptoms as the most consistent cue of hypertension (U) [24, 28]	Perception of taking pills	
Shared cautionary stories of friends impacted by hypertension as sources of motivation (U) [24]		
Perceived sickness only through the guise of physical symptoms (U) [24, 25]		
Perceived symptoms as cues to take medication (U) [28]		
Borrowing NCD medication from colleagues (U) [23]	Coping strategies for maintaining high polypharmacy adherence	
Using home remedies (U) [23]		
Using traditional herbs (U) [23]		
Consulting traditional or faith healers (U) [24]		
Taking both types of medications simultaneously (C) [24]		
Establishing daily reminders (C) [24]		
Involving family members in care (C) [24]		
Having a shared ‘drug bag’ (C) [24]		
View of hypertension as more deadly than HIV (U) [24]	Comparison of the experience of having HIV with having NCDs	7. Some PLWH with NCDs consider hypertension and cancer more concerning conditions than HIV. However, they still describe the ability of HIV to hibernate. Having NCDs may mask concerns regarding HIV and HIV-positive experiences, which can reinforce the self-management of NCDs.
Transmission (U) [24]		
Perception of hypertension as more severe than HIV (U) [24]		
View cancer as a totally different type of disease (U) [25]		
Difference in the survival rates of HIV and cancer (U) [25]		
Identification of cancer as the most concerning condition (U) [25]		
Concerns regarding NCDs that mask concerns regarding HIV (U) [25]		
View of having NCDs with HIV as the same as having NCDs without HIV (U) [31]		
Describing HIV as in hibernation (U) [31]		
View ART resistance as the most feared consequence (U) [24]	Comparison of ARTs with other NCD medication	
Belief that medications for hypertension give PLWH energy (U) [24]		
Belief that medications are necessary for HIV but not for hypertension (U) [24]		
View the effectiveness of ART and antihypertensive medications (U) [24]		
Contradictions between HIV and NCD treatment (U) [25, 26]	Conflicting treatment and information	
Conflicting information (U) [21, 26]		
Reinforcement of self-efficacy for one disease by self-efficacy for another disease (U) [24]	Reinforcement of the self-management of other diseases by HIV	
Encouraging someone else to adhere to treatment based on one’s own experience of ART (U) [24]		
Impact of long-term HIV care on adherence to CVD medication (U) [24, 28]		
Acceptance of the hypertension diagnosis (U) [29]		
Rethinking of the definition of self-management (U) [32]		
Readiness to engage but awareness of limitations due to HIV and NCDs (U) [30]	Exercising	8. While facing HIV with NCDs, PLWH can develop positive coping strategies to accept the realities of living with multiple chronic conditions.
Physical impairments (U) [30]		
Mental health challenges (U) [30]		
Uncertainty (U) [30]		
Social support (C) [30]		
Perceptions and beliefs (U) [30]		
Feeling of accomplishment (U) [30]		
Familial support from either the immediate family or their partner (U) [17, 24]	Seeking family support	
Occasional lending of money by family members to pay for drugs to maintain medication adherence (U) [17, 24]		
Love burden (U) [24]		
Familial support (U) [17]		
Eagerness to share with their friends and/or loved ones for support (U) [17]		
Companionship received from male partners as an inspiration to self-manage HIV and comorbidities (U) [24]		
Simultaneous acquisition of knowledge regarding the disease process and social support (U) [17]	Changing life goals	
Utilization of the available resources to avoid isolation and depression (U) [17]		
Satisfaction with providers (U) [1, 4]		
Likely elimination of the stigma attached to the ARV clinic through the provision of integrated care through general systems (U) [29]		
Frustration with being told by healthcare providers to change lifestyle habits (U) [24]	Making lifestyle adjustments	
Initial period of adjustment with beginning a new medication regimen and new lifestyle changes (U) [24]		
CVD prevention knowledge inconsistent with PLWH CVD risk behavior (U) [28]		
Factors motivating the adoption of and adherence to heart-healthy behaviors (U) [6, 10]	Motivation for adopting healthy behaviors	
Lack of motivation or interest in exercise (U) [30]		
Readiness to engage in exercise as a dynamic construct (U) [30]	Readiness to engage in exercise	
Feeling of readiness to engage in exercise amidst unique circumstances (U) [30]		
Resilience (U) [22]	Developing resilience	
Struggles with tolerating treatment (U) [17]	Developing a self-capacity to manage HIV and comorbidities	
Initiation of learning about the virus from the moment of accepting the diagnosis (U) [17]		
Initiation of learning to be an example for others (U) [17]		
Discordance between providers’ recommendations and the preferred strategy for CVD prevention among PLWH (U) [28]		
Increased ability and desire to self-manage one’s health with age (U) [32]		
Spirituality (U) [27]	Addressing spiritual needs	
Addressing spiritual needs (U) [31]		
From a state of ownership to one of self-advocacy (U) [17]	Increasing subjective initiative	
Self-advocacy for one’s overall health (U) [17]		
Frustration and struggles with new day-to-day routines (U) [17]	Daily struggles	9. Some PLWH living with chronic diseases struggle with new daily routines, emotional difficulties, and family issues. In extreme cases, PLWH may have suicidal ideation when experiencing high pressure.
Existing rather than living (U) [17]		
Emotional and mental health difficulties (U) [22]		
Daily family or personal issues (U) [22]		
Profound psychological effects of physical and social dislocation from the family home on PLWH (U) [18]	Suicide	
Combination of socioeconomic factors, political factors, and chronic illnesses (U) [21]		
Suicidal ideation (U) [25]		

#### Synthesized finding 1

Although the current healthcare systems have many barriers preventing PLWH with NCDs from accessing both HIV and NCD care, they can receive resources and knowledge through healthcare providers.

This synthesized finding originated from 19 findings that were grouped into three categories. These studies described many barriers to accessing healthcare for both HIV and NCDs. Regarding access to NCD healthcare services, the barriers included unavailability of screening machines and/or medications for NCDs [23], mobility restrictions [30], lack of care continuity [29], insufficient care for NCDs [29], and a lack of familiarity with the situations of PLWH among healthcare providers [31]. Regarding access to HIV-related healthcare services, transport costs [23, 30], parking places [22], medication shortages [23, 27], and long waiting times [29] are the main barriers. PLWH’s level of income [23, 30], physical disability [30], and stigma [29] also affect their access to healthcare services.

*“As for me, my blood pressure (BP) was only checked once this year. Most of the time I come here, there will be no electricity, so the BP machine will not be working. Sometimes you come here, and you are told the machine is being used in the maternity ward.” (A participant from South Africa)* [23]

Although barriers were identified, some participants were still satisfied with their providers [17, 22] and the current HIV-related healthcare services in terms of the simultaneous acquisition of knowledge about the disease process and social support [17]. PLWH could utilize available resources to avoid isolation and depression under the current systems [17]. Receiving integrated HIV/NCD care through general systems was likely to remove PLWH’ stigma attached to the antiretroviral therapy clinic [29].

*“They do a lot of tea time; they’ll have workshops on living well with HIV, how to make out your will, how to live on a budget, healthier exercise, healthy eating. So, that’s really good.” (A participant from the United States)* [17]

#### Synthesized finding 2

The factors that can affect care continuity and treatment adherence for both HIV and NCDs among PLWH include the individual physical, mental, and financial statuses of PLWH; their relationships with clinicians; and fragmented healthcare systems.

This synthesized finding stemmed from 15 findings that were grouped into three categories. The factors affecting PLWH’s care continuity and treatment adherence for both HIV and NCDs were categorized as individual factors, fragmented healthcare systems, and patient-clinician partnerships. Individual factors, such as substance abuse [26], mental issues [26], insurance [27], financial status [27], and manifestation of diseases [27], were related to PLWH’s care continuity and treatment adherence. In addition, PLWH’s perception of the consequence of nonadherence to treatment [24, 26] and ease of obtaining prescriptions [23, 26, 27] were major barriers to continuously receiving treatment.

*“[When] I was getting high smoking crack and using drugs, I really didn’t care whether I was HIV-positive and high blood pressure or anything.” (A participant from the United States)* [26]

Among all factors, fragmented and uncoordinated HIV care and chronic care [21, 22, 29, 31] and HIV being beyond generalists’ scope of practice [17, 22, 29] were the most commonly mentioned reasons for why PLWH do not continuously seek healthcare for NCDs.

*“I attend four clinics [ … ]. Today, I am here; then, next month, I’ll attend two other clinics.”* [4]

*“I always get my ARVs from Ntabiseng clinic in Bara and my diabetes pills from the diabetes clinic.” (A participant from South Africa)* [21]

#### Synthesized finding 3

PLWH with NCDs have long-term physical and psychological manifestations of disease. Psychological manifestations are severe and prevalent when PLWH are informed that they have NCDs. Some changes, including changes in appearance and sexual function, can affect individuals’ daily lives, especially among women living with HIV and NCDs.

This synthesized finding was obtained from 16 findings that were grouped into four categories. Studies have noted that PLWH with NCDs have a series of physical and psychological manifestations of disease [17, 21, 22, 24, 29, 32]. The participants reported that having NCDs disrupted their daily life and basic function [17, 27]. However, PLWH with NCDs lack the assumption that any new physical symptoms are related to either HIV or NCDs [31]. Regarding the psychological manifestations, PLWH reported initially having difficulty accepting the diagnosis of multimorbidities and having negative emotions toward the future, such as fear and anger [17, 24]. Especially among PLWH with cancer, the reactions to a cancer diagnosis were similar to the initial reactions to the HIV diagnosis [22]. The participants also feared receiving cancer treatment [22].

*“No one wants to hear that word cancer, that you have it. Now you have this diagnosis, and the first thing you think is okay, death is next. Life is almost over.” (A participant from the United States)* [22]

PLWH with NCDs also reported changes in appearance and sexual function [17, 27, 31]. Women with PLWH and NCDs suffer severe long-term manifestations, including oral health issues, changes in physical appearance, decreased self-esteem, and dyspareunia [17, 32].

*“One of the side effects is that you have light atrophy where the muscle patch in your face disappears, and so suddenly, you're walking around looking like a human skeleton. You look in the mirror and … and I don’t think anyone’s really fond of looking in the mirror at all, just even to comb your hair, brush your teeth. It’s like a passing glance because it just makes you too depressed to see the physicality that’s happened.” (A participant from the United States)* [17]

#### Synthesized finding 4

Having more chronic conditions implies more medical costs for PLWH. The more severe the financial hardship of PLWH, the more negatively it affects their employment and family relationships.

This synthesized finding originated from 10 findings that were grouped into two categories. PLWH reported that having NCDs affected their affordability of medications [17, 23], income [32], and access to health insurance [17, 32]. Having more NCDs implied more medical costs for PLWH. Some PLWH even rely on a minimal state disability grant and lack income [18, 32]. They compared free ART to paid hypertension medication [24].

*“Taking [hypertension] medicine is not a choice but a problem where I cannot find the medicine, while the ART, I have never been in a situation that I want to go get the drugs but find that there is no medicine at the hospital … all times, they are there and are free” (A participant from Malawi)* [24]

From a long-term perspective, having NCDs directly increased the number of hospital appointments [18] and indirectly affected their employment [17, 18], family relationships [18], and confidence [24]. The more severe the financial hardship of PLWH, the more negatively it affected their employment and family relationships.

*“Sometimes it happens that you don’t have money to buy the drugs … with time, if you haven’t done any work and the drugs are almost finished, you don’t know what to do.” (A participant from Malawi)* [24]

#### Synthesized finding 5

PLWH with NCDs experience double stigma toward their HIV and chronic conditions, which may exacerbate their perceived discrimination and lead to social and physical isolation.

This synthesized finding was derived from 12 findings that were grouped into two categories. The categories included double stigma and disease disclosure. Seven studies described double stigma from HIV and NCDs [17, 18, 22, 25, 27, 29, 31]. The stigma was mainly due to having HIV, which may lead to social and physical isolation [18]. However, among these studies, some PLWH reported that a new form of stigma was experienced by PLWH living with cancer [25]. Some PLWH thought that society was a stigmatized environment for people with multimorbidity with HIV [31].

*“They’re trying to make HIV like cancer or anything else. I mean we all kind of wanted that kind of acceptance, but it’s still not as common as cancer or lupus or anything. You know it still has its stigma to it.” (A participant from the United States)* [31]*.*

*“They hid HIV before. But now, they talk about it. Now, they hide cancer. People, they are very negative about cancer.” (A participant from South Africa)* [25]

#### Synthesized finding 6

PLWH with NCDs have high levels of polypharmacy burden for both ART and other medications. This population has difficulty maintaining high medication adherence due to medication fatigue, side effects, and the large numbers of pills to be taken. PLWH with NCDs may spontaneously use some strategies individually or collectively to achieve high levels of medication adherence.

This synthesized finding originated from 22 findings that were grouped into five categories, including pill burden, reasons for taking pills, side effects, polypharmacy adherence, and coping strategies. Among all categories, polypharmacy adherence was regarded as a relatively new problem for PLWH in five articles [17, 23, 24, 26, 32]. Some PLWH with NCDs believed that taking pills daily was a form of survivorship [17]. The side effects and complexity of NCD regimens are regarded as impediments to adherence [24, 26]. The phenomenon of drug fatigue appeared among PLWH with NCDs, who reported difficulty keeping up with the regimens for both HIV and comorbidities [32].

*“One time, I had stopped taking my medicine for a whole month, you know. I’m tired of taking medicine.” (A participant from the United States)* [32]

PLWH with NCDs may spontaneously use some strategies individually or collectively to achieve high levels of medication adherence, such as borrowing NCD medication from colleagues [23], consulting traditional or faith healers [24], taking both HIV and NCD medications simultaneously [24], establishing daily reminders [24], involving family members in care [24], and using a shared “drug bag” [24]. To decrease the financial burden, some PLWH used less expensive NCD remedies (including home remedies and traditional herbs) or resorted to faith and traditional healers instead of using prescribed allopathic medicines. Therefore, they were able to maintain a high level of therapeutic compliance and medication adherence with traditional therapy and remedies [23].

*“I know that [prescribed] drugs are effective … but one needs money to buy them. Prophets, traditional healers … and home remedies are cheaper or even free, … so, I can’t watch myself die when these options are there.” (A participant from South Africa)* [23]

#### Synthesized finding 7

Some PLWH with NCDs consider hypertension and cancer more concerning conditions than HIV. However, they still describe the ability of HIV to hibernate. Having NCDs may mask concerns regarding HIV and HIV-positive experiences, which can reinforce the self-management of NCDs.

This synthesized finding stemmed from 20 findings that were grouped into four categories. Three studies compared the experience of having HIV with NCDs [24, 25, 31]. Some PLWH with hypertension viewed hypertension as deadlier and more severe than HIV [24]. Some PLWH with cancer identified cancer as the most concerning condition [25]. Some participants viewed having NCDs with HIV the same as having NCDs without HIV [31]. Having NCDs masked concerns regarding HIV, but PLWH still described their HIV as in hibernation [25, 31].

*“I got the HIV, I ain’t going to worry about that. I have to worry about having a heart attack or a stroke.” (A participant from the United States)* [26]

PLWH with NCDs compared ART with NCDs treatment. One study from Malawi showed that PLWH considered ART resistance to be the most fearful outcome [24]. Some participants believed that medication for hypertension gave them energy, but other participants believed that medications were necessary for HIV but not for hypertension [24]. Three studies described conflicting treatments and information between their HIV and NCD care [21, 25, 26].

*“Chemo really almost killed me. It was the cause of my white blood cells dying, [the doctors] told me that my CD4 cells are down.” (A participant from the United States)* [26]

Four studies reported that HIV can reinforce the self-management of other NCDs [24, 28, 29, 32]. Long-term HIV care experience had a positive impact on the acceptance of NCD diagnosis and NCD medication adherence [24, 28, 29]. PLWH with NCDs also encouraged others to adhere to treatment based on their experience with ART [24].

#### Synthesized finding 8

While facing HIV with NCDs, PLWH can develop positive coping strategies to accept the realities of living with multiple chronic conditions.

This synthesized finding was derived from 34 findings that were grouped into 10 categories. The positive coping strategies adopted by PLWH with NCDs included exercising [30], seeking family support and social support [17, 24], changing life goals [17, 22, 27], making lifestyle adjustments [24, 28], strengthening motivation to adopt healthy behavior [24, 28, 30], boosting resilience [22], enhancing self-capacity for managing HIV and comorbidities [17, 28, 32], addressing spiritual needs [27, 31], and empowering subjective initiative [17].

*“[Medication] helps my heart; I wanna keep that heart going. I don’t want any heart disease—I don’t have a history of it—but I don’t want to...I have high risk factors, and I try to take care of that with diet, and exercise, and medicine. So, you only get one heart, and it’s okay.” (A participant from the United States)* [28]

#### Synthesized finding 9

Some PLWH living with chronic diseases struggle with new daily routines, emotional difficulties, and family issues. In extreme cases, PLWH may have suicidal ideation when experiencing high pressure.

This synthesized finding originated from seven findings that were grouped into two categories. Some PLWH with NCDs were frustrated and struggled with their new day-to-day routines and family and personal issues [17, 22]. They regarded themselves as simply surviving rather than living [17]. Physical and social dislocation from the family home had profound psychological effects on PLWH [18]. In addition, socioeconomic factors, political factors, and chronic illnesses combined rendered life more challenging [21].

*"My sister had an aneurysm and just drops dead. Then, I said, I can’t do [the cancer treatments] anymore. I’m hurting and I couldn’t, I just said, no, I’m not doing it no more. I stopped everything and I gave up." (A participant from the United States)* [22]

### ConQual summary of the synthesized findings

Table 4 shows the ConQual summary of the synthesized findings. All synthesized findings were downgraded by one level due to dependability limitations. Synthesized findings 2, 3, 6, and 8 were downgraded by one additional level due to a mix of unequivocal and credible findings.

**Table 4:** ConQual summary of the findings

Synthesized Findings	Type of Research	Dependability	Credibility	ConQual Score
1. Although the current healthcare systems have many barriers preventing PLWH with NCDs from accessing both HIV and NCD care, they can receive resources and knowledge through healthcare providers.	Qualitative	Downgrade 1 level	–	Medium
2. The factors that can affect care continuity and treatment adherence for both HIV and NCDs among PLWH include the individual physical, mental, and financial statuses of PLWH; their relationships with clinicians; and fragmented healthcare systems.	Qualitative	Downgrade 1 level	Downgrade 1 level	Low
3. PLWH with NCDs have long-term physical and psychological manifestations of disease. Psychological manifestations are severe and prevalent when PLWH are informed that they have NCDs. Some changes, including changes in appearance and sexual function, can affect individuals’ daily lives, especially among women living with HIV and NCDs.	Qualitative	Downgrade 1 level	Downgrade 1 level	Low
4. Having more chronic conditions implies more medical costs for PLWH. The more severe the financial hardship of PLWH, the more negatively it affects their employment and family relationships.	Qualitative	Downgrade 1 level	–	Medium
5. PLWH with NCDs experience double stigma toward their HIV and chronic conditions, which may exacerbate their perceived discrimination and lead to social and physical isolation.	Qualitative	Downgrade 1 level	–	Medium
6. PLWH with NCDs have high levels of polypharmacy burden for both ART and other medications. This population has difficulty maintaining high medication adherence due to medication fatigue, side effects, and the large numbers of pills to be taken. PLWH with NCDs may spontaneously use some strategies individually or collectively to achieve high levels of medication adherence.	Qualitative	Downgrade 1 level	Downgrade 1 level	Low
7. Some PLWH with NCDs consider hypertension and cancer more concerning conditions than HIV. However, they still describe the ability of HIV to hibernate. Having NCDs may mask concerns regarding HIV and HIV-positive experiences, which can reinforce the self-management of NCDs.	Qualitative	Downgrade 1 level	–	Medium
8. While facing HIV with NCDs, PLWH can develop positive coping strategies to accept the realities of living with multiple chronic conditions.	Qualitative	Downgrade 1 level	Downgrade 1 level	Low
9. Some PLWH living with chronic diseases struggle with new daily routines, emotional difficulties, and family issues. In extreme cases, PLWH may have suicidal ideation when experiencing high pressure.	Qualitative	Downgrade 1 level	–	Medium

## Discussion

This systematic review provides a comprehensive picture of the experiences of PLWH with NCDs, including the perception of fragmented healthcare systems for PLWH receiving NCD care and continuity of accessing both HIV and NCD care. Factors, including manifestations of disease, financial hardship, discrimination and stigma, polypharmacy burden and adherence, also affect PLWH’s daily life coping with NCDs. Some studies provided evidence that PLWH had new viewpoints toward reciprocal relationships between HIV and NCDs since being diagnosed with NCDs. This experience increases PLWH’s initiative and self-capacity for self-management for both HIV and NCDs.

The findings from the meta-aggregation revealed that PLWH with NCDs had a high demand for accessing integrated healthcare services that can link HIV care and NCD care. An integrated healthcare model can not only promote a high level of care continuity among PLWH but also eliminate individuals’ stigma attached to HIV clinics. Previous studies reported several integrated healthcare models of access to NCD care among PLWH [33, 34]. Mwagomba and colleagues analyzed HIV/NCD care integration models in Malawi, South Africa, Swaziland, and Kenya [33]. These authors found that the South African model integrated both HIV and NCDs into primary care and that the other three countries integrated NCD care into HIV specialized clinics; however, these practices did not scale up. Kwarisiima and colleagues conducted a pilot study to integrate HIV care in a chronic disease care model that offered the joint assessment and management of hypertension and diabetes and found that scheduled visit intervals were more frequent among PLWH receiving HIV/NCD care models than among their counterparts [34]. Most available evidence regarding HIV/NCD models was generated from research-based and funded projects. There is a need to conduct more pragmatic trials to implement research findings in a real-world setting. These studies could generate more robust evidence regarding the effects on key HIV and NCD outcomes, cost-effectiveness, and PLWH feedback. Our study provides evidence regarding the perception of HIV/NCD integrated healthcare services among PLWH, which could build a scientific foundation for future interventions [35].

Our study found that PLWH with NCDs had medication fatigue due to polypharmacy. Medication fatigue is a phenomenon in which the habit of taking medication becomes exhausting [36]. This finding is consistent with previous quantitative studies showing that pharmacotherapeutic complexity was a crucial factor for not taking medications [37, 38]. Claborn and colleagues’ systematic review revealed that side effects, patient-provider interaction, treatment intensity, and time since treatment initiation were factors affecting PLWH medication fatigue [36]. In the near future, a single-tablet treatment regimen and long-acting therapies could be resolutions for addressing medication fatigue. In addition, an increased understanding of medication fatigue among PLWH with NCDs is also expected to improve adherence interventions. A systematic assessment of medication fatigue among PLWH with NCDs is needed to address this issue.

In addition, we found that PLWH were more likely to adhere to ART than NCD medication. A reason for this finding may be that ART adherence offered a reduction in the viral load in the short term, but the outcomes of NCD medication, especially prevention medication, may not be immediately effective. This phenomenon also reflects insufficient health education regarding NCD medication among PLWH. Previous studies showed that PLWH did not associate NCD medication adherence with a reduction in severe health conditions and that their knowledge and behaviors related to NCD were discordant [39, 40]. Therefore, healthcare professionals should help PLWH develop strategies to monitor NCD medication adherence once PLWH achieve VS. It is also important to increase the understanding of the logic of NCD treatment plans among PLWH to truly strengthen their self-capacity and participation in care for NCDs [28].

We found six studies reporting that PLWH with NCDs viewed long-term HIV-positive experiences in a more positive light than those with NCDs. They regarded their NCDs as masking their concerns about HIV and perceived these diseases, especially cancer and hypertension, as more severe. This phenomenon can be explained by a consideration of all conditions to be more serious than HIV among PLWH. People who have lived with HIV for a long time are more familiar with HIV than with NCDs, which may lead them to focus on a physically more aggressive condition in the short term or long term [25, 41]. However, the priority of care of PLWH may not be consistent with professionals’ perspective [40]. Interventions for communication regarding how PLWH think about HIV/NCD comorbidities may improve the accuracy of the opinions of PLWH regarding healthy and positive living with NCDs.

Among the included studies, one study from South Africa reported a new form of stigma identified in PLWH with cancer [25]. Nearly one-third of PLWH with cancer stated they would not speak about cancer due to the cultural stereotype that cancer is a fatal disease and a punishment for immoral behaviors. This new form of stigma attached to cancer is context-related. Previous studies reported that the society labeling process finally led to the negative effect of stigma for cancer [42–44]. Oystacher and colleagues found that the society labeling process of cancer stigma occurred through the appearance of perceived signs or symptoms of cancer [42]. In a region with high HIV prevalence, people may conflate the symptomatology of HIV with cancer symptoms. In addition, stereotypes of deadliness associated with both HIV and cancer may contribute to stigmatization. Therefore, education campaigns regarding PLWH living with cancer should be implemented to break the stereotype of cancer. Intervention reducing HIV/AIDS stigma may also lead to a reduction in stigma for cancer.

Some issues need to be considered in the interpretation of our findings. First, the levels of dependability of all 9 synthesized findings were downgraded due to the lack of reporting on the authors’ influences on the studies and lack of information locating the studies culturally and theoretically. We recommend that future qualitative studies strengthen their methodological quality. Second, most included studies were conducted in the US and South Africa. The limited cultural variation may impact how PLWH address NCDs. Therefore, the findings cannot be generalizable to places with diverse contexts. More studies are needed among populations in Asia and Europe.

## Conclusion

This systematic review synthesized qualitative evidence regarding the experience of PLWH with NCDs. The results showed that the current health care systems in the US, Canada, South Africa, Malawi, and Ghana are well-positioned to address the problem of HIV. In recent years, attempts have been made to institutionalize NCD preventive and control services in HIV long-term care. However, considering the growing problem of HIV and NCD comorbidity globally, integrated primary health care systems are needed to address the problems of PLWH with NCDs. The findings supported that an HIV/NCD integrated healthcare service delivery model, such as a model integrating HIV and NCDs into primary care, integrating NCD care into specialized HIV care, or integrating HIV care into NCD care clinics, should be developed to link the fragmented healthcare systems of HIV and NCDs. Healthcare professionals should help PLWH develop strategies to better monitor their polypharmacy burden and adherence, stigma and discrimination, financial hardship, and manifestations of multiple conditions to achieve high levels of care continuity. It is also important to increase the understanding of the logic of NCD treatment plans among PLWH to strengthen their coping strategies and participation in care for NCDs.

## Supplementary Information

**Additional file 1.** Searching strategies and results.

**Additional file 2.** PRISMA Statement.

## Data Availability

The datasets used and/or analyzed during the current study available from the corresponding author on reasonable request.
