# Supplementary material for: Experience of chronic noncommunicable disease in people living with HIV: a systematic review and meta-aggregation of qualitative studies
Source: BMC Public Health. 2021 Sep 10;21:1651. doi: 10.1186/s12889-021-11698-5 (PMC8431942; doi:10.1186/s12889-021-11698-5)
Supplement: Supplementary file 1 — Additional file 1. Searching strategies and results. [file 12889_2021_11698_MOESM1_ESM.docx]

**Appendix I Searching strategies and results**

**PubMed**

Search time: 2020-11-23 15:20

| **Search** | **Query** | **Items found** |
| --- | --- | --- |
| #1 | HIV Infections[MeSH] OR HIV[MeSH] OR Acquired Immunodeficiency Syndrome[MeSH] | 319644 |
| #2 | hiv[tiab] OR hiv infect*[tiab] OR AIDS[tiab] OR PLWHA[tiab] OR PLWH[tiab] OR human immunodeficiency virus[tiab] OR human immunedeficiency virus[tiab] OR human immune-deficiency virus[tiab] OR human immune-deficiency virus[tiab] OR ((human immun*[tiab]) AND (deficiency virus[tiab])) OR acquired immunodeficiency syndrome[tiab] OR acquired immunedeficiency syndrome[tiab] OR acquired immune-deficiency syndrome[tiab] OR acquired immune-deficiency syndrome[tiab] OR ((acquired immun*[tiab]) AND (deficiency syndrome[tiab])) | 416220 |
| #3 | #1 OR #2 | 452606 |
| #4 | Comorbidity[MESH] OR Multimorbidity[MESH] OR Multiple Chronic Conditions[MESH] | 112070 |
| #5 | comorbid*[tiab] OR co-morbid*[tiab] OR multimorbid*[tiab] OR multi-morbid*[tiab] OR multidisease? [tiab] OR multi-disease? [tiab] OR (multiple[tiab] AND (ill*[tiab] OR disease? [tiab] OR condition? [tiab] OR syndrom*[tiab] OR disorder? [tiab])) | 527018 |
| #6 | #4 OR #5 | 590491 |
| #7 | diabetes mellitus[MESH] OR diabet*[tiab] | 710018 |
| #8 | hypertension[MESH] OR hypertens*[tiab] OR "high blood pressure?"[tiab] | 507975 |
| #9 | heart diseases[MESH] OR ((heart[tiab] OR cardiac[tiab] OR cardiovascular[tiab] OR coronar[tiab]) AND (disease?[tiab] OR disorder?[tiab] OR failure[tiab]) OR arrythmia?[tiab]) | 1472634 |
| #10 | cerebrovascular disorders[MESH] OR ((cerebrovascular[tiab] OR vascular[tiab] OR carotoid*[tiab] OR arter*[tiab]) AND (disorder?[tiab] OR disease?[tiab])) | 701680 |
| #11 | asthma[MESH] OR asthma*[tiab] | 181250 |
| #12 | pulmonary disease chronic obstructive[MESH] OR copd[tiab] OR (pulmonary[tiab] AND (disease?[tiab] OR disorder? [tiab])) | 202958 |
| #13 | hyperlipidemia[MESH] OR hyperlipidem*[tiab] OR hypercholesterolemia*[tiab] OR hypertriglyceridemia*[tiab] | 97942 |
| #14 | arthritis rheumatoid[MESH] OR rheumatoid arthritis[tiab] | 149948 |
| #15 | neoplasms[MESH] OR neoplasm?[tiab] OR cancer?[tiab] | 3891313 |
| #16 | kidney diseases[MESH] OR (kidney[tiab] AND (disease?[tiab] or disorder?[tiab])) | 575079 |
| #17 | liver diseases[MESH] OR (liver[tiab] AND (disease?[tiab] or disorder?[tiab])) | 646358 |
| #18 | osteoporosis[MESH] OR osteoporosis[tiab] | 88679 |
| #19 | #7 OR #8 OR #9 OR #10 OR #11 OR #12 OR #13 OR #14 OR #15 OR #16 OR #17 OR #18 | 7664496 |
| #20 | (coocur* or co-occur* or coexist* or co-exist* or multipl*) AND (ill*[tiab] OR disease?[tiab] OR condition?[tiab] OR syndrom*[tiab] OR disorder?[tiab]) | 442851 |
| #21 | #19 AND #20 | 204120 |
| #22 | Qualitative[Title/Abstract] OR experience[Title/Abstract] OR phenomenology[Title/Abstract] OR ethnography[Title/Abstract] OR “grounded theory”[Title/Abstract] OR “mixed method”[Title/Abstract] OR interview[Title/Abstract] OR “focus group”[Title/Abstract] OR diary[Title/Abstract] OR “qualitative research”[MeSH Terms] | 1048642 |
| #23 | #3 AND #6 AND #21 AND #22 Filters: Chinese, English, from 1996/1/1 - | 3139 |

**Medline (Ovid)**

Search time: 2020-11-23 16:48

| **Search** | **Query** | **Items found** |
| --- | --- | --- |
| 1 | (HIV or Acquired Immunodeficiency Syndrome).hw. | 325477 |
| 2 | (hiv or AIDS or PLWHA or PLWH or acquired immunodeficiency syndrome).ti,ab | 358802 |
| 3 | or/1-2 | 406734 |
| 4 | Comorbidity/ | 110634 |
| 5 | (comorbid$ or co‐morbid$).ti,ab. | 151423 |
| 6 | (multimorbid$ or multi‐morbid$).ti,ab. | 4374 |
| 7 | (multidisease? or multi‐disease? or (multiple adj (ill$ or disease? or condition? or syndrom$ or disorder?))).ti,ab. | 4415 |
| 8 | or/4-7 | 225028 |
| 9 | Chronic disease/ | 264815 |
| 10 | (chronic$ adj3 (disease? or ill$ or care or condition? or disorder$ or health$ or medication$ or syndrom$ or symptom$)).ti,ab. | 331202 |
| 11 | or/9-10 | 532198 |
| 12 | 8 or 11 | 729986 |
| 13 | exp diabetes mellitus/ or diabet$.ti,ab. | 617101 |
| 14 | exp hypertension/ or (hypertens$ or "high blood pressure?").ti,ab. | 456700 |
| 15 | exp heart diseases/ or (((heart or cardiac or cardiovascular or coronary) adj (disease? or disorder? or failure)) or arrythmia?).ti,ab. | 1326729 |
| 16 | exp cerebrovascular disorders/ or ((cerebrovascular or vascular or carotoid$ or arter$) adj (disorder? or disease?)).ti,ab. | 501833 |
| 17 | exp asthma/ or asthma$.ti,ab. | 164678 |
| 18 | exp pulmonary disease chronic obstructive/ or (copd or (pulmonary adj2 (disease? or disorder?))).ti,ab. | 100309 |
| 19 | exp hyperlipidemia/ or (hyperlipidem$ or Hypercholesterolemia$ or hypertriglyceridemia$).ti,ab. | 90522 |
| 20 | exp Thyroid diseases/ or ((thyroid adj (disease? or disorder)) or hyperthyroid$ or hypothyroid$).ti,ab. | 159411 |
| 21 | exp arthritis rheumatoid/ or rheumatoid arthritis.ti,ab. | 138071 |
| 22 | exp neoplasms/ or (neoplasm? or cancer?).ti,ab. | 3624514 |
| 23 | exp kidney diseases/ or (kidney adj (disease? or disorder?)).ti,ab. | 530293 |
| 24 | exp liver diseases/ or (liver adj (disease? or disorder?)).ti,ab. | 573183 |
| 25 | exp osteoporosis/ or osteoporosis.ti,ab. | 77038 |
| 26 | or/13-25 | 7083746 |
| 27 | ((coocur$ or co-ocur$ or coexist$ or co-exist$ or multipl$) adj3 (disease? or ill$ or care or condition? or disorder$ or health$ or medication$ or symptom$ or syndrom$)).ti,ab. | 67891 |
| 28 | 26 and 27 | 26559 |
| 29 | 12 or 28 | 750126 |
| 30 | 3 and 29 | 16048 |
| 31 | (Qualitative or experience or phenomenology or ethnography or grounded theory or mixed method or interview or focus group or diary).ti,ab | 876077 |
| 32 | qualitative research.hw. | 58238 |
| 33 | or/31-32 | 886524 |
| 34 | 30 and 33 | 1468 |

**EMBASE (Ovid)**

Search time: 2020-11-23 17:30

| **Search** | **Query** | **Items found** |
| --- | --- | --- |
| 1 | (HIV or Acquired Immunodeficiency Syndrome).hw. | 29212 |
| 2 | (hiv or AIDS or PLWHA or PLWH or acquired immunodeficiency syndrome).ti,ab | 501237 |
| 3 | or/1-2 | 504640 |
| 4 | Comorbidity/ | 281269 |
| 5 | (comorbid$ or co‐morbid$).ti,ab. | 336956 |
| 6 | (multimorbid$ or multi‐morbid$).ti,ab. | 7839 |
| 7 | (multidisease? or multi‐disease? or (multiple adj (ill$ or disease? or condition? or syndrom$ or disorder?))).ti,ab. | 7562 |
| 8 | or/4-7 | 451418 |
| 9 | Chronic disease/ | 179885 |
| 10 | (chronic$ adj3 (disease? or ill$ or care or condition? or disorder$ or health$ or medication$ or syndrom$ or symptom$)).ti,ab. | 567295 |
| 11 | or/9-10 | 668103 |
| 12 | 8 or 11 | 1070515 |
| 13 | exp diabetes mellitus/ or diabet$.ti,ab. | 1160362 |
| 14 | exp hypertension/ or (hypertens$ or "high blood pressure?").ti,ab. | 958008 |
| 15 | exp heart diseases/ or (((heart or cardiac or cardiovascular or coronary) adj (disease? or disorder? or failure)) or arrythmia?).ti,ab. | 2123544 |
| 16 | exp cerebrovascular disorders/ or ((cerebrovascular or vascular or carotoid$ or arter$) adj (disorder? or disease?)).ti,ab. | 857221 |
| 17 | exp asthma/ or asthma$.ti,ab. | 295671 |
| 18 | exp pulmonary disease chronic obstructive/ or (copd or (pulmonary adj2 (disease? or disorder?))).ti,ab. | 198031 |
| 19 | exp hyperlipidemia/ or (hyperlipidem$ or Hypercholesterolemia$ or hypertriglyceridemia$).ti,ab. | 176751 |
| 20 | exp Thyroid diseases/ or ((thyroid adj (disease? or disorder)) or hyperthyroid$ or hypothyroid$).ti,ab. | 239026 |
| 21 | exp arthritis rheumatoid/ or rheumatoid arthritis.ti,ab. | 225315 |
| 22 | exp neoplasms/ or (neoplasm? or cancer?).ti,ab. | 5071486 |
| 23 | exp kidney diseases/ or (kidney adj (disease? or disorder?)).ti,ab. | 971444 |
| 24 | exp liver diseases/ or (liver adj (disease? or disorder?)).ti,ab. | 1024179 |
| 25 | exp osteoporosis/ or osteoporosis.ti,ab. | 152048 |
| 26 | or/13-25 | 10382850 |
| 27 | ((coocur$ or co-ocur$ or coexist$ or co-exist$ or multipl$) adj3 (disease? or ill$ or care or condition? or disorder$ or health$ or medication$ or symptom$ or syndrom$)).ti,ab. | 119965 |
| 28 | 26 and 27 | 54337 |
| 29 | 12 or 28 | 1110964 |
| 30 | 3 and 29 | 24985 |
| 31 | (Qualitative or experience or phenomenology or ethnography or grounded theory or mixed method or interview or focus group or diary).ti,ab | 1422042 |
| 32 | qualitative research.hw. | 81959 |
| 33 | or/31-32 | 1433454 |
| 34 | 30 and 33 | 2404 |

**CINAHL (EBSCO)**

Search time: 2020-11-23 19:04

| **Search** | **Query** | **Items found** |
| --- | --- | --- |
| S1 | (MH HIV+) or (MH "Human Immunodeficiency Virus+") or (MH "Acquired Immunodeficiency Syndrome+") | 26039 |
| S2 | TI (hiv or AIDS or PLWHA or PLWH or human immunodeficiency virus or human immune-deficiency virus or human immunedeficiency virus or human immune-deficiency virus OR acquired immune-deficiency syndrome OR acquired immunedeficiency syndrome or acquired immunodeficiency syndrome or acquired immune-deficiency syndrome) or AB(hiv or AIDS or PLWHA or PLWH or human immunodeficiency virus or human immune-deficiency virus or human immunedeficiency virus or human immune-deficiency virus or acquired immune-deficiency syndrome or acquired immunedeficiency syndrome or acquired immunodeficiency syndrome or acquired immune-deficiency syndrome) | 117794 |
| S3 | S1 OR S2 | 123726 |
| S4 | (MH "Comorbidity") | 62940 |
| S5 | TI (multimorbid* or multi-morbid* or comorbid* or co-morbid* or multidisease? or multi-disease?) or AB (multimorbid* or multi-morbid* or comorbid* or co-morbid* or multidisease? or multi-disease?) or TI (multiple N2 ill* or multiple N2 disease? or multiple N2 condition? or multiple N2 syndrom* or multiple N2 disorder?) or AB (multiple N2 ill* or multiple N2 disease? or multiple N2 condition? or multiple N2 syndrom* or multiple N2 disorder?) or TI (coocur* N3 disease? or coocur* N3 ill*) or AB (coocur* N3 disease? or coocur* N3 ill*) | 79013 |
| S6 | S4 or S5 | 118151 |
| S7 | (MH "Chronic Disease") | 65378 |
| S8 | TI (chronic* W3 disease? or chronic* W3 ill* or chronic* W3 care or chronic* W3 condition? or chronic* W3 disorder* or chronic* W3 health* or chronic* W3 medication* or chronic* W3 syndrom* or chronic* W3 symptom*) or AB (chronic* W3 disease? or chronic* W3 ill* or chronic* W3 care or chronic* W3 condition? or chronic* W3 disorder* or chronic* W3 health* or chronic* W3 medication* or chronic* W3 syndrom* or chronic* W3 symptom*) | 115834 |
| S9 | S7 or S8 | 158356 |
| S10 | (MH "Diabetes Mellitus+") | 168802 |
| S11 | (MM "Hypertension+") OR (MM "Cerebrovascular Disorders+") | 141625 |
| S12 | (MM "Cardiovascular Diseases+") | 497796 |
| S13 | (MM "Lung Diseases, Obstructive+") OR (MM "Pulmonary Disease, Chronic Obstructive+") OR (MM "Asthma+") | 48753 |
| S14 | (MM "Thyroid Diseases+") | 13992 |
| S15 | (MM "Arthritis+") | 60143 |
| S16 | (MM "Epilepsy+") | 14608 |
| S17 | (MM "Liver Diseases+") | 57193 |
| S18 | (MM "Neoplasms+") | 185919 |
| S19 | (MM "Osteoporosis+") | 15874 |
| S20 | (MM "Kidney Diseases+") | 68037 |
| S21 | TI diabet* or asthma* or chronic or disease | 1481623 |
| S22 | S10 or S11 or S12 or S13 or S14 or S15 or S16 or S17 or S18 or S19 or S20 or S21 | 2211528 |
| S23 | TI ( coocurr* or coexist* or co-ocurr* or coexist* or co-exist*) or AB (coocurr* or coexist* or co-ocurr* or coexist* or co-exist*) | 12149 |
| S24 | S9 and S23 | 1041 |
| S25 | S22 and S23 | 7762 |
| S26 | S24 or S25 | 7765 |
| S27 | S26 and S3 | 178 |
| S28 | TI (Qualitative or experience or phenomenology or ethnography or grounded theory or mixed method or interview or focus group or diary) or AB (Qualitative or experience or phenomenology or ethnography or grounded theory or mixed method or interview or focus group or diary) OR MH "qualitative research" | 527975 |
| S29 | S27 and S28 | 143 |

**Web of Science**

Search time: 2020-11-23 20:40

| **Search** | **Query** | **Items found** |
| --- | --- | --- |
| #1 | TOPIC: (HIV OR Acquired Immunodeficiency Syndrome OR AIDS OR PLWHA OR PLWH OR human immunodeficiency virus OR human immuno‐deficiency virus OR human immunedeficiency virus OR human immune‐deficiency virus OR acquired immune‐deficiency syndrome OR acquired immunedeficiency syndrome OR acquired immunodeficiency syndrome OR acquired immuno‐deficiency syndrome) | 907112 |
| #2 | TOPIC: (comorbid* OR co-morbid* OR multimorbid* OR multi-morbid* OR multidisease? OR multi-disease? OR (multiple AND (ill* OR disease? OR condition? OR syndrom*OR disorder?))) | 572937 |
| #3 | TOPIC: (diabet* OR hypertens* OR "high blood pressure?" OR ((heart OR cardiac OR cardiovascular OR coronar) AND (disease? OR disorder? OR failure)) OR arrythmia? OR ((cerebrovascular OR vascular OR carotoid* OR arter*) AND (disorder? OR disease?[tiab])) OR copd OR (pulmonary AND (disease? OR disorder?)) OR hyperlipidem* OR hypercholesterolemia* OR hypertriglyceridemia* OR rheumatoid arthritis OR neoplasm?[tiab] OR cancer? OR (kidney AND (disease? or disorder?)) OR (liver AND (disease? or disorder?)) OR osteoporosis) | 2311121 |
| #4 | TOPIC:  ((coocur* or co-occur* or coexist* or co-exist* or multipl*) AND (ill* OR disease? OR condition? OR syndrom* OR disorder?)) | 579171 |
| #4 | #2 OR (#3 AND #4) | 597310 |
| #5 | #1 AND #4 | 19315 |
| #6 | TOPIC: (Qualitative OR experience OR phenomenology OR ethnography OR "grounded theory" OR "mixed method" OR interview OR "focus group" OR diary) | 2736392 |
| #7 | #5 AND #6 | 3022 |

**ProQuest Dissertations and Theses (Health & Medical Collection)**

Search time: 2020-11-23 11:04

| **Search** | **Query** | **Items found** |
| --- | --- | --- |
| #1 | ti(HIV OR Acquired Immunodeficiency Syndrome OR AIDS OR PLWHA OR PLWH OR human immunodeficiency virus OR human immuno‐deficiency virus OR human immunedeficiency virus OR human immune‐deficiency virus OR acquired immune‐deficiency syndrome OR acquired immunedeficiency syndrome OR acquired immunodeficiency syndrome OR acquired immuno‐deficiency syndrome) OR ab(HIV OR Acquired Immunodeficiency Syndrome OR AIDS OR PLWHA OR PLWH OR human immunodeficiency virus OR human immuno‐deficiency virus OR human immunedeficiency virus OR human immune‐deficiency virus OR acquired immune‐deficiency syndrome OR acquired immunedeficiency syndrome OR acquired immunodeficiency syndrome OR acquired immuno‐deficiency syndrome) | 302173 |
| #2 | ti(comorbid* OR co-morbid* OR multimorbid* OR multi-morbid* OR multidisease? OR multi-disease? OR (multiple AND (ill* OR disease? OR condition? OR syndrom*OR disorder?))) OR ab(comorbid* OR co-morbid* OR multimorbid* OR multi-morbid* OR multidisease? OR multi-disease? OR (multiple AND (ill* OR disease? OR condition? OR syndrom*OR disorder?))) | 232118 |
| #3 | ti(diabet* OR hypertens* OR "high blood pressure?" OR ((heart OR cardiac OR cardiovascular OR coronar) AND (disease? OR disorder? OR failure)) OR arrythmia? OR ((cerebrovascular OR vascular OR carotoid* OR arter*) AND (disorder? OR disease?[tiab])) OR copd OR (pulmonary AND (disease? OR disorder?)) OR hyperlipidem* OR hypercholesterolemia* OR hypertriglyceridemia* OR rheumatoid arthritis OR neoplasm?[tiab] OR cancer? OR (kidney AND (disease? or disorder?)) OR (liver AND (disease? or disorder?)) OR osteoporosis) OR ab(diabet* OR hypertens* OR "high blood pressure?" OR ((heart OR cardiac OR cardiovascular OR coronar) AND (disease? OR disorder? OR failure)) OR arrythmia? OR ((cerebrovascular OR vascular OR carotoid* OR arter*) AND (disorder? OR disease?[tiab])) OR copd OR (pulmonary AND (disease? OR disorder?)) OR hyperlipidem* OR hypercholesterolemia* OR hypertriglyceridemia* OR rheumatoid arthritis OR neoplasm?[tiab] OR cancer? OR (kidney AND (disease? or disorder?)) OR (liver AND (disease? or disorder?)) OR osteoporosis) | 1992700 |
| #4 | ti((coocur* or co-occur* or coexist* or co-exist* or multipl*) AND (ill* OR disease? OR condition? OR syndrom* OR disorder?)) OR ab((coocur* or co-occur* or coexist* or co-exist* or multipl*) AND (ill* OR disease? OR condition? OR syndrom* OR disorder?)) | 200226 |
| #5 | #3 AND #4 | 58342 |
| #6 | #2 AND #5 | 46823 |
| #7 | #1 AND #6 | 1477 |
| #8 | ti(Qualitative OR experience OR phenomenology OR ethnography OR "grounded theory" OR "mixed method" OR interview OR "focus group" OR diary) OR ab (Qualitative OR experience OR phenomenology OR ethnography OR "grounded theory" OR "mixed method" OR interview OR "focus group" OR diary) | 380072 |
| #9 | #7 AND #8 | 107 |

CNKI^*^

Search time: 2020-11-24 11:30

| **Search** | **Query** | **Items found** |
| --- | --- | --- |
| #1 | [(title, abstract, and keywords)% "Human Immunodeficiency Virus + Acquired Immunodeficiency Syndrome + HIV/AIDS + HIV + AIDS](https://kns.cnki.net/KNS8/AdvSearch?id=54&dbcode=SCDB&searchtype=gradeSearch&ishistory=1) | 111900 |
| #2 | (title, abstract, and keywords)%chronic disease + diabetes +hypertension + COPD + cardiovascular disease + cancer | 1515200 |
| #3 | (title, abstract, and keywords)% qualitative study + interview + grounded theory + ethnography | 1002100 |
| #4 | #1 AND #2 AND #3 | 177 |

*The search strategy was translated into English.

Wanfang^*^

Search time: 2020-11-24 11:50

| **Search** | **Query** | **Items found** |
| --- | --- | --- |
| #1 | (Topic: (HIV OR AIDS OR Human Immunodeficiency Virus OR Acquired Immunodeficiency Syndrome OR HIV/AIDS)) *Date:1996-2020 | 406293 |
| #2 | (Topic: (chronic disease OR diabetes OR hypertension OR COPD OR cardiovascular disease OR cancer)) *Date:1996-2020 | 2590699 |
| #3 | (Topic: (qualitative study OR interview OR grounded theory OR ethnography)) *Date:1996-2020 | 1054295 |
| #4 | #1 AND #2 AND #3 | 132 |

*The search strategy was translated into English.
